# Trajectories of Health Status during the Transition from School to University: Piloting the Method of Biographical Mapping in German Medical Students

**DOI:** 10.3390/children8080622

**Published:** 2021-07-22

**Authors:** Katharina Diehl, Jana Lindenthal

**Affiliations:** Mannheim Institute of Public Health, Social and Preventive Medicine, Medical Faculty Mannheim, Heidelberg University, Ludolf-Krehl-Straße 7-11, 68167 Mannheim, Germany; jana.lindenthal@stud.uni-heidelberg.de

**Keywords:** students, university, health status, trajectories, transition

## Abstract

Health status and health behavior change during the transition from school to university. However, it is still unclear whether these changes occur at specific points in time, and whether these changes are stable. Therefore, our aim was to conduct a pilot test on biographical mapping (BM) for the first time in this research area in order to map the trajectories of the health status of university students over time. This enabled us to also test the practicability of BM, and to assess the agreement of the findings of BM with those of a standardized questionnaire. We included 30 fourth-year university students. First, they filled in a standardized questionnaire on their sociodemographic information, current health status, and health status compared with that for their final year of school. Second, they filled in a BM grid that allows for drawing the changes in health status that have taken place over the transition period. The health status changed during the transition (e.g., slight decline in general subjective health), and was related to specific events before and after the transition (e.g., examinations), showing that all health variables were not stable over time. The findings of BM were also reflected in the standardized questionnaire. Using BM revealed the changes in health during a six-year-period, including the school–university transition. The identified changes in health during transition and at specific time points underline that not only assistance before the transition, but also psychological support during studies, seems to be important for the health promotion of students. Besides this, BM seems to be a useful, although time-consuming, instrument for which the results were similar to those in the questionnaire.

## 1. Introduction

The transition from school to university is a crucial phase for many young people worldwide. The transition is associated with various structural, social, and psychological changes that have an impact on the assumptions, relationships, roles, and routines of these young adults [1,2]. University students become more independent from their parents, their autonomy increases, and they face new sets of responsibilities [3,4,5,6]. Their relationship with their family members may change, and new relationships will develop as many students change their domicile at the start of their studies [7,8].

Previous research shows that the transition to the university level is associated with significant changes in health-related risk behaviors. Some studies have identified a significant increase in the consumption of marijuana and alcohol among university students compared with when they were in high school [9]. Besides this, the transition to the university level is associated with an increase in body weight, as found in several studies [10,11,12,13]. Likewise, unhealthy eating habits become more prevalent [12], especially when students have to stay far from home [14,15]. It has been found that physical activity among university students in many countries is often insufficient [16]. However, not all studies showed changes in frequency [12]. In Germany, where the current study took place, a nationwide survey identified changes in eating behavior and physical activity among university students compared with their previous habits during their last years at school [7,15].

Previous findings have suggested that changes in health behavior take place as part of this transition. As health behavior is strongly related to health status, it can therefore be hypothesized that changes in health behavior might be associated with changes in health status during the transition from school to university. For instance, as exercise and physical activity are beneficial for physical and mental health [17], the lower level of physical activity seen in university students compared with high school students [18] could potentially lead to lower health level. It has been shown in other populations that transitions can be associated with changes in health status, for instance transition to parenthood [19] and transitioning to retirement [20].

Regarding the transition from school to university, the exact time and phase that these potential changes in health status occur, and whether they are constant over time, remains unclear. In research, changes in health or health-related risk behavior before and after the transition from school to university are frequently surveyed by one questionnaire [7]. However, this questionnaire does not cover the trajectories that take place in between the transition, and it remains unclear if the health status or behavior after the transition remain stable over time. To better understand this, longitudinal studies would be helpful, yet they are cost-intensive. An alternative could be using the biographical mapping method. It has been used, for instance, in sports sciences to investigate the development of health status over time [21,22]. Biographical mapping may help to identify and describe the trajectories, transitions, and turning points in health.

The aim of this manuscript is two-fold. First, we aimed to identify whether the health status of university students is stable during their transition from school to university, or whether there is variation. To describe trajectories and identify the turning points in health status, we used a biographical mapping grid. Second, we aimed to compare the results of the biographical mapping grid with standardized questionnaire data gathered at the same time. The underlying aim was to identify whether both approaches reached the same result.

## 2. Materials and Methods

The participants were medical students in their fourth year of study. We used a convenient sample of n = 30 out of about 240 medical students that were currently in their fourth study year. We decided to include only students from one subject and the same year, because they all passed through the same curriculum and had their main life events (i.e., abitur certificate, start of studies, and examinations) in the same time period.

Students were recruited via notifications on the notice board at the university library, social media posts, and direct contact prior to and during university courses. The survey was conducted face-to-face by one of the authors (J.L.), which included two parts: a questionnaire and a mapping grid. Data collection took place from 18 June 2019 to 22 July 2019. All participants gave written informed consent. The study was approved by the Ethics Committee II of the Medical Faculty Mannheim, Heidelberg University (16 April 2019, number 2019-650N).

### 2.1. Questionnaire

The questionnaire included sociodemographic information (e.g., sex, age, and marital status) and university-relevant characteristics (e.g., change of domicile to begin the course). In addition, their current health status was surveyed. We asked, “How would you describe your general health status?”, as well as their “mental health” and “physical health”. The categories of responses to these three questions were very good/good/moderate/bad/very bad. Regarding life satisfaction, we asked, “With my life in general I am…”, and provided seven response options ranging from very satisfied (1) to very unsatisfied (7).

To get an idea of how health status changes compared with high school, we included questions on the retrospectively perceived change. Regarding general, mental, and physical health, we asked, “Compared to the upper secondary level, how would you describe your general/mental/physical health?”, with the response categories of “It has improved”, “It has worsened”, and “It is steady”. The same questions were included regarding changes in general life satisfaction. These items are based on a quantitative nationwide study among university students [6].

### 2.2. Mapping Grid

After having filled in the questionnaire, the mapping grid was introduced to the participants and was filled in during qualitative interviews conducted by J.L. (doctoral student). Two grids were printed on A4-sized, landscape-oriented sheets of paper for each interview. One grid focused on health-related risk behavior, while the other grid focused on health status over time. The focus of this manuscript is on health status.

Five different-colored pens were provided for the participants to draw their different health status trajectories (i.e., self-rated general health, self-rated mental health, self-rated physical health, general life satisfaction, and perceived stress). The interviewer explained to the participants that the x-axis of the mapping grid reflects the timeline for the past years, starting from 2013. The y-axis reflects a scale from 0 (very bad) to 10 (very good).

**Figure 1:**
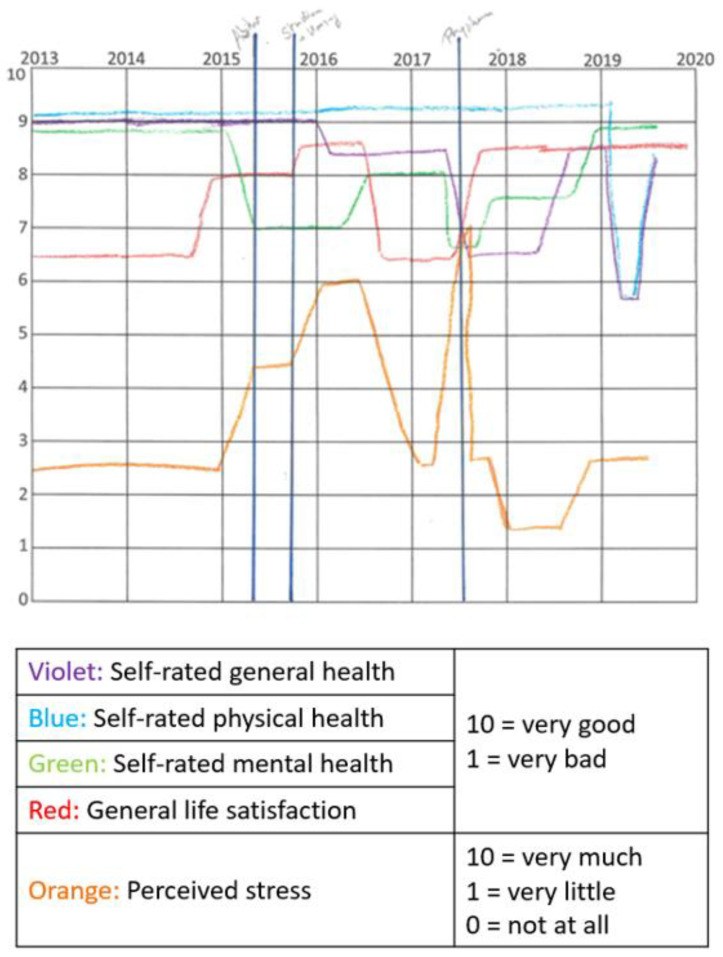
Example of a filled in mapping grid.

In the first step, they marked important events that were relevant to all participants (“abitur certificate”, “start of studies”, and “preliminary examination”). Besides this, they could add important personal events, such as separation from the partner or death in the family. In the second step, using five different-colored pens, they drew individual lines for each health outcome. While drawing the trajectories, they were guided by the a priori marked life events and the years that were displayed at the upper side of the mapping grid. During both steps, the interviewer guided and assisted the participants. An example of a filled in mapping grid on health status can be found in Figure 1.

### 2.3. Analysis

First, we used univariate statistics to describe our study population using the responses to the questionnaire. To get an idea of the health status and the changes in the health status after transitioning to university compared with the health status at school, we used uni- and bivariate statistics and Fisher’s exact test.

Second, to analyze the mapping grid, we transferred the numbers on the grid for each quarter of the year into our SPSS data set. To identify the correct number, we used a conventional ruler. We then calculated the mean value of all of the students for each health outcome for each time point. The results were then transferred into figures.

Third, we combined the data from the questionnaire with the data from the mapping grid. We compared the group mean of the last entry in the mapping with the four health variables, which indicate the current health status in the questionnaire. For testing the significance, we used the Kruskal–Wallis H-tests.

Fourth, we calculated the delta of the first and the last entry in the mapping grid for each health outcome, and again calculated the aggregated means that described the difference in health status between the first and the last entry. We then used the responses in the questionnaire on change in health status (improved/worsened/steady) to calculate the group means, including the Kruskal–Wallis-H. All of the analyses were performed using IBM SPSS Statistics Version 27. A *p*-value < 0.05 was considered to be significant.

## 3. Results

Thirty medical students in their fourth year of study participated in our study. The mean age was 22.1 years, and 66.7% were female. Half of the participants (50.0%) had a steady partner. The majority (93.3%) had changed their residence to begin their study at the university.

### 3.1. Results of the Questionnaire

The majority of students showed a good or very good self-rated general, mental, and physical health based on the questionnaire (Table 1). Only a few students reported having moderate health, while none reported having bad or very bad health. Most of the students were satisfied with their general life.

When considering the changes in the students’ health status compared with their high school years, as assessed in the standardized questionnaire (Table 1), we could see differences between the health variables. While 30% of the participants reported worsened general health, and 33% reported worsened physical health compared with the upper secondary level, the proportion was smaller for mental health (17%). In addition, 10% indicated that their general life satisfaction declined, while 57% reported an improved life satisfaction. Significant findings between change in health and current health status were found for self-rated mental health (*p* = 0.008); among those with moderate health, the majority (n = 4, 80%) reported that their mental health worsened compared with when they were in high school, while the majority of those with good and very good mental health reported a stable health status.

### 3.2. Results of the Mapping Grid

**Figure 2:**
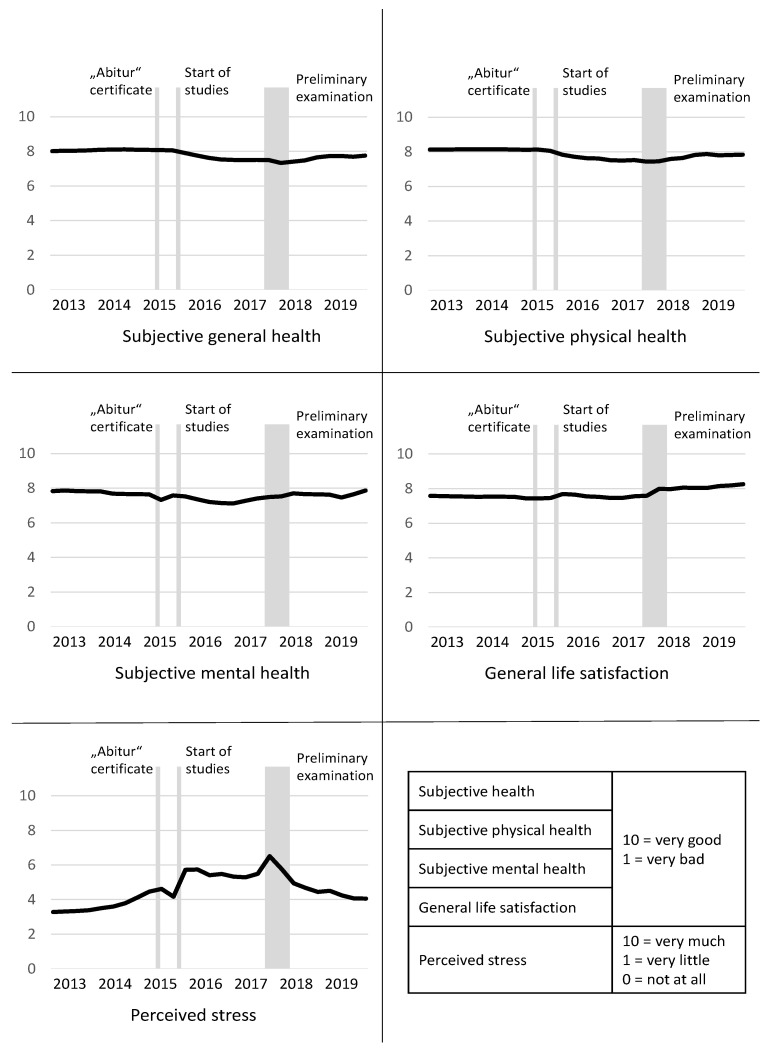
Results of the mapping grid (mean of 30 medical students).

Figure 2 shows the aggregated results of the 30 mapping grids. The mean value for each point in time (i.e., each quarter of a year) is reported. The self-rated general, mental, and physical health status showed a similar level at the beginning and end of the time period. However, we could see changes during the six-year period that were related to events such as abitur certificate, start of studies, and preliminary exams. The mean values for general life satisfaction confirm the increase found in the standardized questionnaires. The hypothesis that specific events are associated with health is also shown for perceived stress, which peaked during the three above-mentioned events. Furthermore, for the life-events that are shown in Figure 2, individuals named separation from partner and death of a reference person as stressors.

### 3.3. Combining the Results from the Questionnaire and the Mapping Grid

Bringing together the current health status from the mapping (last mean value) and the current status of self-rated health and life satisfaction in the questionnaire reveals that the group mean reflects the responses to the questions of the questionnaire: the better the self-rated health, the higher the group mean (Table 2). For instance, regarding self-rated mental health, students who indicated a very good status in the questionnaire had the highest mean in the mapping grid (8.86), followed by those with good health (7.82) and those with moderate health (6.05). This association between the results of the questionnaire and the mapping grid was statistically significant (*p* = 0.003). Regarding general life satisfaction (*p* = 0.007), we found a small inconsistency—while those who were very satisfied in the questionnaire showed the highest mean in the mapping grid (9.30) and those who were satisfied showed the second highest (8.31), those who were neither satisfied nor dissatisfied had a higher mean (7.45; n = 2) than those who were rather satisfied (6.54; n = 4).

Similarly, the change in health status and life satisfaction (i.e., the difference between the first and last value in the mapping grid) and the responses to the comparative questions in the standardized questionnaire match each other (except for the variable on self-rated physical health (n.s.); Table 3). The highest mean delta is shown for those who indicated that their health/satisfaction improved, while the lowest mean delta is shown for those who indicated that their health/satisfaction worsened. For example, those who indicated in the questionnaire that their general health improved compared with their school years showed a mean increase of 0.83 in the mapping grid, while those with a declined health status in the questionnaire had a mean decrease of −1.77 in the mapping grid (*p* < 0.001). The findings were not significant for self-rated mental health (*p* = 0.090), but the association shows the same direction as found for general health and life satisfaction.

## 4. Discussion

This is the first time that biographical mapping was used to map the health status over the transition period from the school to the university. We found changes in the health status that were related to the transition itself, but could also see that the health variables were associated with specific events during the course of their studies, such as examinations. Biographical mapping was proven to be a useful yet time-consuming way of identifying health status over time retrospectively. The results of the mapping grid were also reflected in our questionnaire data based on single-item questions on current and retrospective health status.

### 4.1. Health during the Transition from School to the University

Regarding the different dimensions of health, we found that the majority had a good or very good self-rated general, mental, and physical health. However, the mapping grid revealed that there was variation over time. We could gather more knowledge on the association between health status and the transition from school to university, on the trajectories of health, and the association with specific events.

Regarding self-rated general and physical health, we found a similar course: both decreased after the start of the studies, and increased to a lower level after the preliminary examination until the last entry. Previous research has shown a decrease in physical activity among university students, an increase in substance consumption such as marijuana and alcohol, and weight gain especially in first year students [9,10,11,12,13,23], which might explain the decrease in subjective (physical) health over time. German data have shown that university students are more likely to show worse subjective health compared with same-aged non-students [24].

Our findings suggest that self-rated general, physical, and mental health worsen directly after the start of studies, which demonstrates an important point of contact for prevention. Based on this finding, it seems plausible to introduce some form of preparation for studying at university into the school timetable so as to ascertain whether potential future students would be able to meet the requirements for becoming university students.

However, regarding subjective mental health, we found a different course compared with general and physical health, as it seems to be particularly associated with specific events—the mapping grids show a decrease during “Abitur” examination and after the start of the studies, and an increase after the completion of the preliminary examination. Data comparing German university students with non-students revealed that students are more likely to have mental health problems [25,26,27]. A study conducted in Spain and an earlier study undertaken in the UK confirmed these results [18,28]. The findings that university students often seem to suffer from mental health problems, and that these problems seem to occur in stressful situations, underline the importance of psychological assistance in the campus.

The finding on mental health is even stronger when focusing on stress, which showed peaks during abitur, start of studies, and preliminary examination. During these phases, the students might be particularly vulnerable. Wilkes et al. [29] showed that more medical students meet a professional as a result of their poor mental health during their studies compared with at the start of their studies. They found that 83% of medical students reported that their studies were a significant source of stress [29]. As stress seems to occur in phases, there is therefore a need for the provision and availability of short-term mental health services and counselling.

For general life satisfaction, we found an increase over time in the mapping grid and in the questionnaire. Similar to the previous findings regarding the life satisfaction of students in the medical field, most of the students were satisfied with their general life [30,31]. The mapping grids revealed that the students were much more satisfied with their life at the time point of the survey compared with their last years at school. The explanation for this could be the increase in autonomy and independence that they experienced after finishing school [3,4,5], which might have increased their self-confidence and therewith a higher life satisfaction.

### 4.2. Use and Practicability of the Mapping Grid

The results of the mapping grid on the current health status and change of health status compared with the school years largely correspond with the results of the questionnaire. The mapping grids undeniably entail a lot of additional information compared to the single-item questions of the questionnaire, yet both, to a certain extent, validate each other. However, as other researchers have already described, there is great potential in using a mapping grid to describe health status or health behavior over time [21]. It is very useful for visualizing the development of health-related outcomes over time. Our study showed that the use of mapping grids for transitions might uncover new information on the stability of the health status. In addition to this, it is possible to consider individual events that occurred during the analyzed period. However, analyzing the mapping grids is very time-consuming.

### 4.3. Strengths and Weaknesses

In our study, we combined the quantitative questionnaire with a comparable new method of using biographical mapping embedded in a short qualitative interview. Biographical mapping helped us to visualize the health status over the transitional period from school to the university, which is important in order to better understand the feelings and needs of university students. In the mapping grid, however, we have not been able to give the specific time point of “preliminary examination”, and only refer to it as a phase in which all examinations took place, due to the German system. Nonetheless, it gave a first reference of time. Besides this, we only included 30 medical students, which did not allow us to generalize our results because of the sample size and the specific field of study. However, the case number was sufficient to pilot biographical mapping in this area of research and to test feasibility. In addition, biographical mapping is a qualitative method that is very complex in its conduct and analysis. However, the sample is quite low for the statistical analysis, which may explain the inconsistencies found for the findings on general life satisfaction. A limitation might be the inclusion of students from only one field of study in the same study year. However, this approach made our data better comparable, because they all passed through the same curriculum and had their main life events (i.e., abitur certificate, start of studies, and examination) at the same time points. Nonetheless, future studies should also focus on students from other subjects in order to be able to draw comparisons. A bias that could not be excluded is that students might have had problems in recalling their health status correctly. However, the mapping grid allowed them to think about the development over time, which might have made it easier for them to recall their health status at a specific point in time.

## 5. Conclusions

Although it is a reduction of knowledge, the assessment of health across validated health status scales and the question of current health status compared with an earlier point in time seems to reflect the respective numbers of the mapping well. This means that the use of an elaborate mapping grid in written questionnaires is not absolutely necessary, as the comparative questions would usually reflect the longtime change well. However, the use of the mapping method is suitable in qualitative studies and studies with a small number of cases as it can depict the trajectories of health status caused by special events. This is particularly interesting when—as in this case—the focus of the research is on transitions in which the state of health seems to be rather unstable.

Regarding the health status of the students, it became visible in the mapping grid that an increase in stress and a decrease in mental health are related to events such as important examinations. This underlines the importance of providing mental support that is available in the short-term for students on the campus. The finding that general and physical subjective health declined after starting their studies shows that, first, a better preparation for future studies regarding the demands of university life is necessary in the last years at school, and second, the provision and availability of continuous support (e.g., mentoring, introduction weeks, counselling) is important for helping students stay healthy. Here, biographic mapping would be the method of choice for exploring whether support actually makes a difference, by comparing students who received such support with those who did not. Biographical mapping has the potential to help identify further attachment points for health promotion and prevention in university students.

## Figures and Tables

**Table 1:** Current health status of medical students and change in health status compared with their last year in high school based on the questionnaire.

		Change Compared to High School			
	n (%)	Improved n (%)	Worsened n (%)	Steady n (%)	*p*-Value
Self-rated general health		9 (30%)	9 (30%)	12 (40%)	0.694
Very good	11 (37%)	4	2	5	
Good	16 (53%)	5	5	6	
Moderate	3 (10%)	0	2	1	
Bad	- (-)	-	-	-	
Very Bad	- (-)	-	-	-	
Self-rated mental health		6 (20%)	5 (17%)	19 (63%)	0.008
Very good	9 (30%)	1	0	8	
Good	14 (47%)	3	1	10	
Moderate	7 (23%)	2	4	1	
Bad	- (-)	-	-	-	
Very bad	- (-)	-	-	-	
Self-rated physical health		11 (37%)	10 (33%)	9 (30%)	0.571
Very good	9 (30%)	4	2	3	
Good	17 (57%)	7	6	4	
Moderate	4 (13%)	0	2	2	
Bad	- (-)	-	-	-	
Very bad	- (-)	-	-	-	
General life satisfaction		17 (57%)	3 (10%)	10 (33%)	0.094
Very satisfied	7 (23%)	5	0	2	
Satisfied	17 (57%)	10	1	6	
Rather satisfied	4 (13%)	2	0	2	
Neither satisfied nor dissatisfied	2 (7%)	0	2	0	
Rather dissatisfied	- (-)	-	-	-	
Dissatisfied	- (-)	-	-	-	
Very dissatisfied	- (-)	-	-	-	

n = 30 medical students; *p*-values based on Fisher’s exact test.

**Table 2:** Last mean value in mapping regarding health status by responses to the questionnaire.

	Mapping Grid:	Last Mean Value in Mapping (July 2019)				
		Self-Rated General Health	Self-Rated Mental Health	Self-Rated Physical Health	General Life Satisfaction	
Questionnaire:						
		(7.83)	(7.72)	(7.86)	(8.25)	*p*-Value
Self-rated general health						0.119
Very good		8.23				
Good		7.85				
Moderate		6.25				
Self-rated mental health						0.003
Very good			8.86			
Good			7.82			
Moderate			6.05			
Self-rated physical health						0.152
Very good				8.22		
Good				7.80		
Moderate				7.28		
General life satisfaction						0.007
Very satisfied					9.30	
Satisfied					8.31	
Rather satisfied					6.54	
Neither satisfied nor dissatisfied					7.45	

n = 30 medical students; reported are group means; *p*-value is based on Kruskal—Wallis-H-tests. Values on mapping grid: 10: very good, 1: very bad. Reading assistance: Those who indicated having very good self-rated general health in the questionnaire had a higher last mean value regarding self-rated general health (=8.23) compared with those who indicated to have a good (=7.85) or moderate (=6.25) self-rated general health in the questionnaire.

**Table 3:** Change in health status reported in the mapping grid by responses in the questionnaire.

	Mapping Grid:	Self-Rated General Health	Self-Rated Physical Health	Self-Rated Mental Health	General Life Satisfaction
Questionnaire		(−0.23)	(−0.28)	(−0.16)	(0.64)
Improved		0.83	−0.07	0.62	1.49
Worsened		−1.77	−0.23	−2.12	−1.62
Steady		0.13	−0.59	0.12	−0.13
		*p* < 0.001	*p* = 0.480	*p* = 0.090	*p* = 0.001

n = 30 medical students; reported are group means; *p*-value is based on Kruskal–Wallis-H tests. Change is the difference between the last and the first value in the mapping grid. Reading assistance: In the mapping grid, the mean delta of self-rated general health for the first and the last entry was −0.23. Those who indicated in the questionnaire that their general health improved since school had a mean delta of 0.83, and therefore an increase in the mapping grid. Those who indicated that their general health worsened in the questionnaire showed a mean delta of −1.77 (indicating a decrease in health).

## Data Availability

Data are available from the corresponding author upon reasonable request.
